# Skin Tear Treatment with *Copaifera multijuga* Hayne in Polymeric Hydrogel: A Randomized Clinical Trial

**DOI:** 10.3390/ph17121691

**Published:** 2024-12-15

**Authors:** Camila Castanho Cardinelli, Jéssica Teixeira Gâmba Passos, Valdir Florêncio Veiga-Junior, Beatriz Guitton Renaud Baptista de Oliveira, Elisabete Pereira dos Santos, Guilherme Guilhermino Neto, Karina Chamma Di Piero, Zaida Maria Faria de Freitas

**Affiliations:** 1 Department of Drugs and Medicines, Faculty of Pharmacy, Federal University of Rio de Janeiro, Rio de Janeiro 21941-902, Brazil; 2 Department of Chemical Engineering, Military Institute of Engineering, Rio de Janeiro 22290-270, Brazil; 3 Department of Fundamentals of Nursing and Administration, Federal Fluminense University, Niterói 24020-091, Brazil; 4 Federal Institute of Espírito Santo, Cariacica 29150-410, Brazil; 5 Clementino Fraga Filho University Hospital, Federal University of Rio de Janeiro, Rio de Janeiro 21941-617, Brazil

**Keywords:** controlled clinical trial, wound healing, *Copaifera*, clinical nursing research, rvidence-based nursing, skin tears

## Abstract

A double-blind, randomized, and controlled clinical trial with therapeutic intervention was performed at a university hospital in Rio de Janeiro to evaluate whether the addition of *Copaifera multijuga* Hayne oleoresin to a carboxypolymethylene hydrogel is more effective in skin tear healing than standard treatment. The sample consisted of 84 patients, predominantly men, with a mean age of 67.37 years. These participants were divided into three groups (29 in the intervention group, which received 10% Copaifera oleoresin; 28 in the intervention group, which received 2% Copaifera oleoresin; and 27 in the control group, which received carboxypolymethylene hydrogel). Data were tabulated and analyzed according to the relevant protocols and included only patients who had completed the treatment, while losses were excluded. Weekly follow-ups were conducted to monitor progress. The average healing time differed among the three groups (*p* > 0.05). There was also a significant difference in healing time between the two intervention groups. Ultimately, CopaibaPolyHy-2 led to significantly faster wound healing than CopaibaPolyHy-10 (*p* < 0.05). A high increase in granulation and epithelial tissue and a decrease in exudate quantity were observed in the CopaibaPolyHy-2 group. It was not possible to infer whether the wound size reduction differed between the treatments (*p* > 0.05). At the end of the study, 100% of wounds were healed, with 47,6% healing in week 2 (n = 40). No participants experienced local or serious adverse effects throughout the study period. The current study shows that CopaibaPolyHy-2 is effective, offering a statistically significantly faster healing time, better-quality tissue, and safe treatment for skin tears.

## 1. Introduction

Skin tears (STs) are traumatic wounds that are common among the elderly due to the fragility of their skin, caused by a loss of collagen. Such tears occur due to a combination of friction, shear, and/or blunt force, or just friction [1,2], and are common among white people and women. STs occur mainly in the upper and lower limbs [3,4] and can be complex and painful, sometimes becoming chronic. STs can also affect patient quality of life due to risks of hospitalization [5].

The dressings considered ideal for this etiology should mainly offer a low incidence of friction, shear, and adhesion. The most commonly cited examples in the literature are high-cost foams [6]. A common low-cost option for treatment is hydrogel because of its potential to absorb exudates due to the presence of polymeric chains [7]. Due to the great financial impacts of ST treatments, more accessible resources must be evaluated to optimize treatment follow-up.

From this perspective, research on phytomedicines has increased. In Brazil, interest in Amazonian flora has progressively increased due to its potential in the development of various health care products, as well as its technological potential. The *Copaifera multijuga* Hayne oleoresin (CMOR) product extracted from the Copaibeira tree is known for its potential medicinal activities such as anti-inflammatory and healing effects [8,9,10]. This oleoresin consists of resinous acids and volatile compounds and has been used for medicinal treatments due to its terpenes (copalic acid, β-caryophyllene, and α-copaene), which have anti-endemic, anti-inflammatory, antibacterial, insecticidal, antifungal, and healing effects [8,9,11].

Current treatments are costly. To resolve this issue, phytomedicine based on carboxypolymethylene hydrogel containing CMOR, which has healing and anti-inflammatory potential, could be a low-cost option for treatment. The aim of this study was to evaluate the healing times and reduction sizes of STs treated with polymeric hydrogel formulations containing 2% copaiba oleoresin (CopaibaPolyHy-2) and 10% copaiba oleoresin (CopaibaPolyHy-10) compared with the effects when using polymeric hydrogel with no active ingredient (control). The primary outcome was the healing time, and the secondary outcome was the reduction in wound area.

## 2. Results

### 2.1. Prepare and Evaluation of Polymer Hydrogels

The main components found in CMOR are the sesquiterpene β-caryophyllene (34.75%) and the diterpene copalic acid (6.46%). The action of Copaifera is recognized in the literature mainly due to the presence of its terpenes. β-caryophyllene is associated with healing potential, as sesquiterpene is often associated with anti-inflammatory, anti-edematous, and bactericidal activities [8,12,13]. The main diterpene, copalic acid, is known to have potential applications in protecting against external agents, in addition to its antibacterial potential [14,15].

The evaluated organoleptic aspects were as expected. All formulations had a milky white appearance, characteristic odor, consistent texture, and good spreadability. Density also remained within expectations. These associated factors are fundamental to the success of wound treatment, as they enable treatment to remain within established limits, without macerating the edges and hindering treatment progression.

The composition of the control formulation was 3.13% polysorbate 80, 1.87% sorbitan monoleate 80, 0.10% methylparaben, 10% glycerin, 0.80% carboxypolymethylene, 0.70% 2-amino-2-methyl-1-propanol, and distilled water, for a total of 100 g. For the CopaíbaPolyHy-2 and CopaíbaPolyHy-10 formulations, respectively, we added 2% and 10% CMOR. All formulations were prepared using the classic gel preparation method [16].

The pH assessment also remained within expectations, with both formulations presenting levels between 6.90 and 7.10. A basic pH helps to reduce pain, since the expected pH level for intact human skin is between 4.00 and 6.00, and the wound bed has a pH value closer to neutrality, which can be altered by endogenous and exogenous factors.

The formulation remained stable, without separation of the aqueous and oil phases, during stability tests at temperatures of 40 °C, 60 °C, and 80 °C, with centrifugation at 6400 rpm/10 min per temperature interval. The microbiological control results for the polymeric hydrogels also remained within expectations, without any changes.

According to the results obtained for both the physicochemical properties and the microbiological tests, all formulations presented suitable characteristics for the topical treatment of wounds.

The development of a new semi-solid formulation, such as CopaibaPolyHy, that is easy to prepare and could be safe and effective for the treatment of wounds represents an important therapeutic advance. The formulation’s applicability is further supported by its national economic viability.

### 2.2. Clinical Trial Results

**Figure 1:**
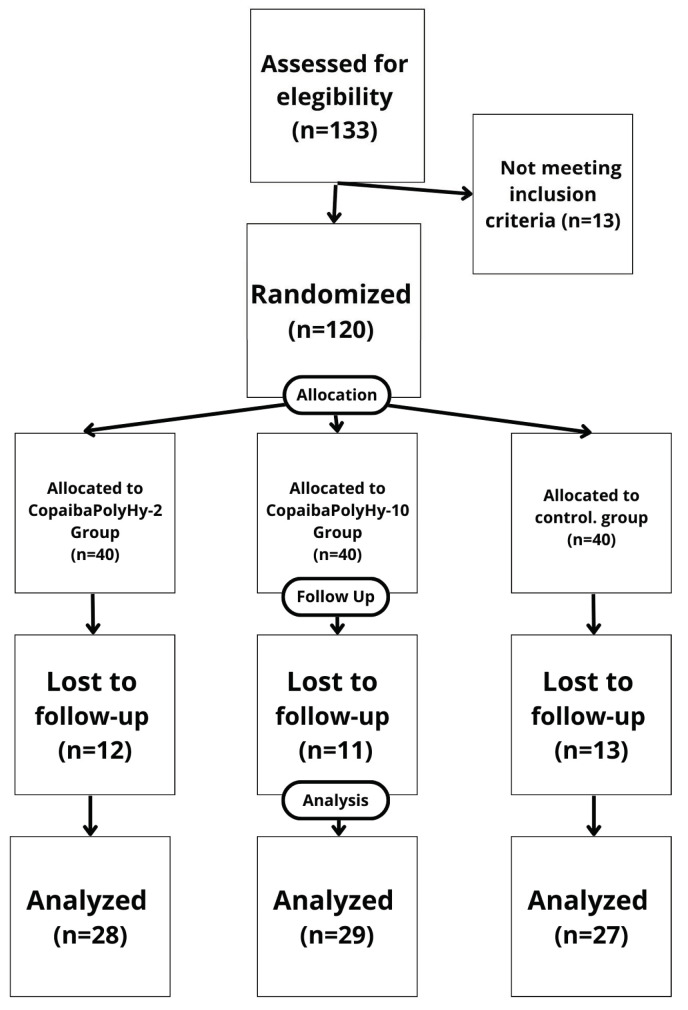
CONSORT diagram.

The sample was equally distributed (*p* > 0.05) with 28 participants in the CopaibaPolyHy-2 intervention group, 29 in the CopaibaPolyHy-10 intervention group, and 27 in the control group (Figure 1).

Each participant attended 2 to 10 follow-up nursing consultations weekly, totaling 300 assessments. The participants were monitored until their wounds were fully healed. In total, 133 participants were selected through screening, but only 120 participants were included in the study based on the inclusion criteria. Thirteen of the participants did not meet the inclusion criteria and were not available to attend weekly appointments. Data collection took place between January 2022 and February 2023.

### 2.3. Baseline Data

The participants were aged between 21 and 92 years old, with an average age of 67.37 years. There was no statistical difference between the groups evaluated (*p* < 0.05); the mean for CopaibaPolyHy-2 was 65.57 years (SD ± 13.66); that for the control was 68.27 years (SD ± 14.77); and that for CopaibaPolyHy-10 was 68.27 years (SD ± 13.62).

Males were predominant in the study in all groups (54.16%). The most common underlying diseases were systemic arterial hypertension (70%), diabetes mellitus (39.16%), and chronic kidney disease (14.16%).

The most commonly affected topographies in all groups were the upper and lower limbs (78.34%) due to the fragility of the extremities. STs here are often caused by the presence of psychomotor modernity, a loss of sensitivity, and weakness. There was no difference in wound areas between the three groups.

The findings of the present study highlight polypharmacy as an important risk factor since only two patients did not use continuous medicines, and more than 90% used two or more medicines. The most common classes were hypoglycemic medicines, gastric protectors, anticoagulants, and antihypertensives.

### 2.4. Primary Outcome—“Healing Time”

Therapeutic outcomes for the 120 participants were mapped throughout the treatments with CopaibaPolyHy-2, CopaibaPolyHy-10, and the control. During the treatment, 36 patients were excluded due to a loss of follow-up. In total, 84 participants were considered for the analysis of the outcomes. For participants who continued treatment, there was no significant difference (*p* > 0.05) in healing outcomes. All 84 patients treated from beginning to end achieved complete healing. In this sense, we could infer a 100% healing rate.

The first variable analyzed was the time required to achieve healing, measured in days, with the aim of evaluating whether there was a significant difference (*p* > 0.05) between treatments (Table 1).

The boxplot presents the distribution of time until complete healing. The analysis suggests similar results in the CopaibaPolyHy-2 and control treatments (*p* > 0.05). The group treated with CopaibaPolyHy-10 differed, showing longer mean and median times, greater standard deviation (suggesting less predictability in relation to expected time), and one patient whose time was reasonably longer than the others (62 days).

Since the CopaibaPolyHy-10 group appeared to have worse performance than the others, ANOVA was used for analysis of variance. In this way, five outliers were found and excluded from the analysis, including two from the control group (28 and 35 days), one from the CopaibaPolyHy-2 group (29 days), and one from the CopaibaPolyHy-10 group (62 days).

The normality of the sample was determined using a QQ-plot for each group in relation to the analyzed variable. The analysis demonstrated that the data followed a normal distribution in each group. This evidence was corroborated by the results of the Shapiro–Wilk test for each group, i.e., the control (*p* = 0.0946), CopaibaPolyHy-2 (*p* = 0.1740), and CopaibaPolyHy-10 (*p* = 0.0711) groups. Based on a significance level *α* = 0.05, it can be assumed that the data were normally distributed (*p* > 0.05).

After testing the homogeneity of variances using a Levene test (*p* = 0.3920), we found no significant difference in the variances between the three groups. Moreover, the ANOVA results were *F* = 3.432, *p* = 0.037, and generalized *η*2 = 0.082. This provided sufficient evidence at a significance level of 0.05 to reject the hypothesis that the average time, in days, until healing would be equal between the groups (*p* < 0.05). Therefore, 8.2% of the variation in time in days may be due to differences in treatments.

Additionally, a Tukey test was performed. The result obtained between the control group and CopaibaPolyHy-2 group was *p* = 0.8170, whereas the result obtained between the control group and the CopaibaPolyHy-10 group was *p* = 0.1590. Lastly, the result obtained between the CopaibaPolyHy-2 group and the CopaibaPolyHy-10 was *p* = 0.0368. The only significant difference found was between the CopaibaPolyHy-2 group and the CopaibaPolyHy-10 group, where the result was *p* < 0.05.

The average time for ST healing was described according to the classification of tissue damage in the studied STs. Wounds with no tissue loss (type 1) required an average of 9.83 days to completely heal, while wounds with partial tissue loss (type 2) required 19 days. In cases of total tissue loss (type 3), the estimated time was 21 days. Our findings show that type 1 wounds required 6.25 days to heal, type 2 wounds required 12.35 days, and type 3 wounds required 12.61 days.

### 2.5. Secondary Outcome—“Reduction in Wound Area”

The second variable analyzed was the reduction in wound area in in^2^/day (cm^2^/day) since the wounds presented in significantly different areas. The averages found for the treatments were 0.355 inches for the control group, 0.399 inches for the CopaibaPolyHy-2 group, and 0.331 inches for the CopaibaPolyHy-10 group. The minimum and maximum areas for each group were 3.2700 inches and 0.0145 inches for the control group, 3.9300 inches and 0.0364 inches for the CopaibaPolyHy-2 group, and 1.4300 inches and 0.0154 inches for the CopaibaPolyHy-10 group. Further, the interquartile ranges were 0.239 inches in the control group, 0.201 inches in the CopaibaPolyHy-2 group, and 0.444 inches in the CopaibaPolyHy-10 group. A new box plot was generated based on these data.

Moreover, both analyses indicated greater variability in the results for CopaibaPolyHy-10. Meanwhile, the variability was smaller for CopaibaPolyHy-2, with an interquartile range of half the amplitude, in addition to a greater and smaller median reduction, respectively, with CopaibaPolyHy-2 and CopaibaPolyHy-10.

The Shapiro–Wilk test indicated a non-normal distribution in all groups (*p* < 0.05). Consequently, the Kruskal–Wallis test was performed as an alternative to ANOVA. The *p* value in the Kruskal–Wallis test was approximately *p* = 0. Therefore, we can reject the hypothesis that the average reduction in wound area (in in^2^/day (cm^2^/day)) would be equal between the groups. After testing this difference, the Wilcoxon test did not indicate the existence of a difference in the average reduction in wound areas between groups (*p* > 0.05).

During wound treatment, an average of five grams of hydrogel was used in dressings for wounds measuring up to 8.06 in^2^/day (5 cm^2^). The CopaibaPolyHy-10 dressings were composed of 0.03% copalic acid and 0.17% β-caryophyllene, while the CopaibaPolyHy-2 dressings were composed of 0.06% copalic acid and 0.03% β-caryophyllene.

The mechanism of action for each active element of Copaifera oleoresin has not yet been fully elucidated; therefore, it is not possible to make further inferences.

### 2.6. Harm

There was no serious or local adverse events such as pain, itching, or eczema during follow-up.

## 3. Discussion

The topical treatment of skin tears is fundamentally important to their clinical evolution, especially among the elderly. The age range highlighted corroborates the evidence that the elderly are most frequently affected by these wounds. This factor made age a relevant factor in this research. The older the age, the greater the exposure to risk factors related to aging [17].

The effects of CopaibaPolyHy on wound healing were observable. The complex mixture of sesquiterpenes and diterpenes forms a complex synergy, which may be related to the advantage of using CopaibaPolyHy-2 over CopaibaPolyHy-10. Lower concentrations may be more likely to achieve a balance between the aforementioned factors [18], and the fibroblasts and collagen fibers appeared to be more organized. This result might be associated with faster contraction of the injured tissue [19,20].

The presence of β-caryophyllene in the oleoresin composition is one of the main advantages of wound re-epithelialization. This sesquiterpene can increase the distance between keratinocytes in the intact perilesional skin towards the center of the wound, which indicates improved re-epithelialization. In other words, the cells at the edge of the wound proliferated more easily and favored formation of the extracellular matrix (ECM) on the granulation tissue. Copalic acid, a diterpene found in greater quantities in oleoresin, is associated with antibacterial potential, which favors healing, preventing the lesion from being contaminated by external agents [14,21,22].

Although *Copaifera* may have cytotoxic properties, especially in high doses, its effects vary depending on its use [23]. The absence of adverse reactions in the current study indicates that the concentrations used may be safe for in vivo applications.

Moreover, the use of oleoresin showed an increase in activated macrophages, which are responsible for modulating the inflammatory process, protecting tissue, and vascular proliferation. Increased vascularization can cause a delay in the proliferative phase as it can increase the inflammation time of the wound [24].

**Figure 2:**
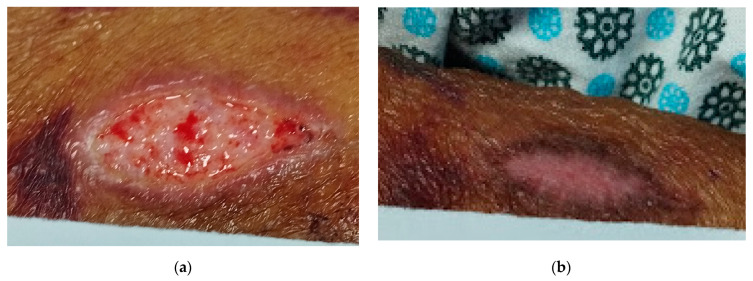
(**a**) An ST treated with CopaibaPolyHy-10 on the 15th day; (**b**) the same ST healed on the 32nd day.

The characteristics of the two different concentrations showed that they can directly influence wound remodulation. In the macroscopic evaluation (Figure 2), the tissues of wounds dressed with CopaibaPolyHy-10 presented granulation that was irregular and opaque, similar in appearance to hyper granulation. This aspect of hyper granulation caused the healing process to stall for some time, delaying the wound remodeling process. Therefore, this treatment option was less predictable than the others. However, it was not possible to infer whether the treatment was solely responsible for the healing time. Systemic factors may also be related to this delay.

Additionally, this tissue factor could be related to the fact that a higher percentage of oleoresin in the formulation corresponds to a faster healing process. Nevertheless, this process is not always ideal for the wound itself [25]. Although the result was not significantly better than that of CopaibaPolyHy-2 (*p* > 0.05), the wounds treated with the control still achieved a good healing outcome, with observable linear treatment of the lesion tissue. This characteristic is directly influenced by the use of polymeric hydrogels. The treatment that offered the greatest advantage with a significant difference (*p* < 0.05) was CopaibaPolyHy-2. This result occurred because even if large portions of the wounds treated using CopaibaPolyHy-10 had already re-epithelialized, necrotic tissue was still found in the wound bed.

Despite the high concentration, the results of this study highlight copaiba’s anti-inflammatory effects, as well as its stimulation of angiogenesis, re-epithelialization, retraction of wound edges, and removal of the extracellular matrix. Oleoresin’s impact on proliferating fibroblasts and collagen synthesis is influenced by the greater expression of FGF-2 and could also be related with the tissue favoring the crust, thus increasing the stratified epithelium and vascularization of the wounds [15,26,27]. Copaiba may be related to a decrease in the release of these inflammation mediators or receptor blockade, resulting in peripheral antinociceptive effects [28].

## 4. Materials and Methods

### 4.1. Methodology

The present study was a randomized parallel double-blind comparative clinical trial conducted between January 2022 and February 2023. Eighty-four individuals from a federal university hospital in Rio de Janeiro, Brazil, who presented with STs participated in this study.

### 4.2. Ethical Considerations

This study was performed in accordance with the ethical principles of the Declaration of Helsinki and good clinical practice recommendations. The study was approved on 20 August 2020 by the research ethics committee of the university at which the study took place (Opinion No. 4.225.729). The study was also registered in the Brazilian Registry of Clinical Trial (RBR-10xhkk8c). Participants confirmed their acceptance by reading and signing the consent form.

### 4.3. Participants

The target population included patients over 18 years of age. The registered nursing staff initially identified potential participants based on wound etiology and provided them with an information sheet to demonstrate their interest in participating. The researcher then provided complete details of the study and obtained informed written consent from each participant.

The main inclusion criterion was the presence of a shallow ST less than 2 inches in area, preferably in the upper or lower limbs, while the exclusion criteria were patients using vasoactive drugs, those unable to understand the study protocol, those with allergies to copaiba, and those with necrotic tissue in the wound or clinical signs of wound infection. The discontinuation criteria were non-adherence to the treatment protocol, the presence of serious adverse events, or the death of the patient during evaluation. Furthermore, if an eligible participant had more than one appropriate ST, only one ST was selected for the purposes of the study.

### 4.4. Randomization

Eligible patients were randomized using a computer-based randomization program and blinded to the researcher. For this process, block randomization was carried out, and patients were coded according to their order of acceptance into the treatment group or control group, as determined by the software (40 patients for each). The 120 patients were divided into three groups: two using treatments (CopaíbaPolyHy-2 and CopaíbaPolyHy-10) and the control group. The control group received a formula of hydrogel with no active ingredient. The primary endpoint included the proportion of healed STs during the period of the study. The secondary endpoint involved those for which the wound size changed each week. Weekly assessments included measuring wound sizes, evaluating pain, and determining any adverse events.

### 4.5. Sample Size

The sample size calculation was performed with a significance level *α* = 0.05. To estimate the population mean and the variance of time until recovery, we used *μ* = 12.5 days and *σA*2 = *σZ*2 = 66 = 36 days^2^, respectively. Such data were previously noted in a study on wound healing [29].

This study is the first randomized control trial (RCT) using copaiba. Consequently, there is also no RCT available to compare healing time. The sample was composed of 120 patients, with 40 in the CopaibaPolyHy-2 group, 40 in the CopaibaPolyHy-10 group, and 40 in the control group.

### 4.6. Wound Dressing

The CMOR was acquired from the Aripuanã Guariba Agroextractivist Association and supplemented by a technical report. The CMOR was then duly characterized by the Analytical Center of the Federal University of Amazonas using Gas-Phase Chromatography coupled with Mass Spectrometry. The project was registered in the National Genetic Heritage Management System (SISGEN)—A43A234.

The polymeric hydrogels CopaibaPolyHy-2 and CopaibaPolyHy-10 and the control used in the clinical trial were produced and evaluated at the Semisolid Laboratory of the University Pharmacy of the Federal University of Rio de Janeiro (UFRJ) according to best practices [16,30]. A 22-pound batch was produced for each of the three treatments over the entire study. We analyzed the physical–chemical properties in all treatments (organoleptic aspects and determination of pH and density [31]; stability at different temperatures [16]; and microbiological control (Standard Plate Count; Molds and Yeasts, *Pseudomonas aeruginosa*, *Clostridium*, *Staphylococcus aureus*, Bac. Gram-neg., bile-tolerant) [32]. Microbiological analyses were carried out at the Medicines, Food, and Cosmetics Quality Control Laboratory at the Faculty of Pharmacy of UFRJ.

The interventions used were CopaibaPolyHy-2, CopaibaPolyHy-10, and a control according to the protocol established in the study. The protocol consisted of cleaning the wound with 0.9% saline solution, drying the edges, applying CopaibaPolyHy-2, CopaibaPolyHy-10, or the control, and covering the wound with sterile gauze and adhesive. After the intervention, participants received an informative instrument with all written instructions to repeat the process at home.

The three formulations were kept in 50 g or 100 g tubes to avoid contamination and were identical in their presentation to ensure the blinding of the study. The label contained the place and date of manufacture, and the code used to blind the products. The formulas had codes for identification. In addition, colored stickers (orange—CopaibaPolyHy-2; gold—control; and black—CopaibaPolyHy-10) were added to facilitate use and understanding of the blinded treatment among the patient and family. The researcher, participants, and statistical analyses were blinded to the treatments.

### 4.7. Wound Healing

Disposable paper rulers were used to measure the longest wound length and width dimensions to provide the estimated wound surface area. The STs were classified into types 1, 2, or 3 according to the ISTAP classification system [33]. The use of a cross-culturally validated and adapted tool in Portuguese was essential for health professionals to carry out the most appropriate categorization of this etiology and construct future epidemiological data [34].

Healing rates and the percentage of wound area reduction were calculated using the proportionate changes in mean surface area. All subjects were evaluated weekly from week 0 (the start) until fully healed (at the end of the study). Wound photographs were taken weekly during the time of the assessment. The Bates–Jensen Wound Assessment Tool was used to monitor the progression of STs.

### 4.8. Data Analysis

The quantitative analysis was carried out by a statistician external to the research group to ensure blinding. The data were stored in a password-protected Microsoft Excel 2020 spreadsheet. Using the data collected, we carried out an exploratory analysis of the variables of interest. These analyses studied the healing time and reduction in wound area using summary measures (such as positional and dispersion measurements), frequency tables, contingency tables, and data visualization tools. The data analysis was carried out according to the relevant protocol. Thus, we included only participants who received the treatment and were initially fully allocated. In this analysis, first, the significant difference in the average healing time in days between treatments was tested. Then, an analysis of variance (ANOVA) [35] was conducted for the first time.

Here, *μ*days*A*, *μ*days*B*, and *μ*days*C* represent the average times for the CopaibaPolyHy-2, CopaibaPolyHy-10, and control groups, respectively. The null hypothesis (*H*0) was that the average time for days until healing among the three treatments would be equal, while the alternative hypothesis (*H*1) was that at least one of the treatment groups would have a different average number of days until healing. The significance level adopted was 0.95. When we were unable to reject the null hypothesis, a Tukey test [36] was performed to compare the means of independent samples, pair by pair, to identify where among the groups the differences were located.

We also monitored the day-over-day evolution in the same group regarding the size of the wound area. Based on *δA*, *δB*, and *δC* being the average difference in wound area (using in^2^/day (cm^2^/day)), the following hypotheses were tested via ANOVA: *H*0: *δA* = *δB* = *δC*; *H*1: At least one group has a different mean. In cases where it was not possible to reject the null hypothesis, Wilcoxon tests [36] were performed to identify which groups were different from each other.

## 5. Conclusions

The results of this clinical trial demonstrated that the treatment of STs with CopaibaPolyHy-2 was statistically better (*p* > 0.05) than treatment with CopaibaPolyHy-10 in accelerating healing, with a significant reduction in wound area. This outcome suggests that CopaibaPolyHy-2 is not only safe but also more effective and affordable than the other concentration tested.

The development of this phytomedicine with copaiba could effectively improve tissue repair. Future research should evaluate the cost-effectiveness of subsequent operational protocols for the treatment of patients with STs in the public health system.

## Figures and Tables

**Table 1:** Descriptive statistics for time (in days) until complete healing by treatment.

Treatment	Average Healing Time	Minimum Time	Maximum Time
CopaibaPolyHy-2	10.0 days	2 days	29 days
CopaibaPolyHy-10	14.6 days	4 days	62 days
Control	11.7 days	3 days	35 days

## Data Availability

The original contributions presented in this study are included in the article. Further inquiries can be directed to the corresponding authors.
